# The Struggle for Legitimacy in Business and Human Rights Regulation—a Consideration of the Processes Leading to the UN Guiding Principles and an International Treaty

**DOI:** 10.1007/s12142-020-00612-y

**Published:** 2021-01-05

**Authors:** Brigitte Hamm

**Affiliations:** Institute for Development and Peace (INEF), Duisburg, Germany

**Keywords:** Business and human rights, Legitimacy, UN Guiding Principles, Business and human rights treaty, Governance, Multi-stakeholder initiatives

## Abstract

After the UN Guiding Principles on Business and Human Rights (UNGPs) were adopted in 2011, an international treaty has been being negotiated since 2014. The two instruments reveal similarities and also conflicts regarding the adequate organization of the global economy based on human rights. The focus in this article will be on the processes leading to these instruments, because they themselves mirror different understandings of governance in the field of business and human rights as well as the struggle over the power of definition and legitimacy. The UNGPs were developed on the basis of global multi-stakeholder consultations, underlining legitimacy through broad inclusion. There are varying judgements as to the success of this approach. The process towards the treaty follows the traditional path of negotiations at UN level. These negotiations reveal a struggle for recognition of the legitimacy of the process itself. Both procedures have shortcomings with regard to legitimacy and show the need for a revision concerning the inclusion of stakeholders. The complementarity of a soft and hard law instrument may enhance the creation of a level playing field in the global economy, thereby strengthening human rights.

## Introduction

When the *UN Guiding Principles on Business and Human Rights* (UNGPs) were unanimously endorsed by the UN Human Rights Council (HRC) in June 2011, this was a big step towards strengthening corporate responsibility for human rights in the global economy (HRC 2011; Office of the High Commissioner 2011). Transnational corporations (TNCs) from different sectors welcomed the new instrument. The same is true for governments especially from Western industrial countries, and the European Union (EU) turned out to be a big supporter of the UNGPs. However, already at the time of adoption, some critical voices pointed to shortcomings with respect to process and content. Thus, the portrayal of the so-called Ruggie process as a comprehensive and successful multi-stakeholder approach (Aaronson and Higham 2013) is critically scrutinized by some international law experts such as Carlos López (2013) and Surya Deva (2013). To López (2013: 69f), the consultations were not negotiations, and he also complains about the lack of inclusion of people directly affected by big business projects and victims of corporate human rights abuses. In their controversy with John G. Ruggie, Deva and Bilchitz (2014) resume this issue asking that indigenous peoples’ rights as well as environmental rights should have been included more prominently. Moreover, López classifies the unanimous endorsement of the Guiding Principles by the HRC only as a weak approval as it was reached without a vote, because otherwise Ecuador would have denied consent. López (2013: 70) suggests that at this meeting, the major concern was legitimacy, because the adoption “[…] provided a crucial level of political legitimacy from the UN for a document that would otherwise be without much consequence.” To him, it could never reach a status in international law equivalent to being negotiated among governments. Nevertheless, at the time of adoption, only few downright critical voices were heard such as that of Human Rights Watch: “In effect, the council endorsed the status quo: a world where companies are encouraged, but not obliged, to respect human rights.”^1^

With the 2019 draft of a legally binding instrument on business and human rights (official name: *‘Revised Draft’ of a Legally Binding Instrument to Regulate, in International Human Rights Law, the Activities of Transnational Corporations and other Business Enterprises* (RDLBI)), we have a second instrument at UN level that intends to direct businesses, especially TNCs, to follow human rights standards in the global economy. What is the background for two initiatives on the same topic? Why are the UNGPs perceived by some as being not sufficient? This article will show that we are experiencing a struggle over the power of definition and legitimacy in the field of business and human rights. Definition refers to determining the content of the corporate responsibility for human rights and its implementation. Legitimacy concerns the approval for the adequate mode of governance in the field of business and human rights. This conflict can be perceived as being more fundamentally about the acceptance of the neoliberal course of globalization, emphasizing market solutions and voluntary commitments, when it comes to the responsibility of enterprises towards the society and the environment.

Looking at the processes of how these two instruments evolved (with respect to the RDLBI, how it evolved up to 2019), fundamental differences attract the attention. On the one hand, the UNGPs emerged from a broad multi-stakeholder process with public consultations and conferences all over the world. The claim is that the UNGPs inter alia draw their legitimacy from this broad inclusionary process. In contrast, the RDLBI is elaborated by an open-ended intergovernmental working group (OEIGWG) of the United Nations (UN) with formal procedures, and limited stakeholder inclusion that follows predefined rules. Here, the authority of states and of the UN are major sources of legitimacy.

For the discussion, first the concepts of political steering and of (global) governance with respect to human rights as well as legitimacy under conditions of globalization will be introduced. Before presenting the processes of elaborating the UNGPs and the RDLBI, a short summary of the content of these two instruments seems appropriate, because understanding commonalities and differences provides a better insight into the views dominant in the debate.

## Context of the Business and Human Rights Discourse

After World War II, the adoption of the UN Charter in 1945 marked the beginning of a new international order based on international law and the UN as the leading international organization. One fundamental principle of this order is laid down in Article 2 of the Charter emphasizing the sovereign equality of the UN member states (Article 2 (1)), and non-interference in inner affairs (Article 2 (4)).^2^ To date, the UN has 193 member states, thus representing more or less the world community of states.

Regarding the economy, the postwar years were initially marked by the evolution of a liberal international economic order that until the end of the 1980s was marked by the Cold War and US hegemony (Peterson 2008). Since then, the intensifying integration of the global economy due to free trade and foreign direct investments is striking, and multinational enterprises (MNEs) or TNCs drive this globalization process.^3^ Their number has increased dramatically over the years: While there existed roughly 7000 TNCs in 1970, the United Nations Conference on Trade and Development (UNCTAD) estimated for the year 2007 some 79,000 TNCs with about 790,000 affiliates abroad.^4^ A total of 80% of global trade has become linked to global production networks of TNCs.

Dan Danielsen (2020) captures this situation as supply chain capitalism, emphasizing structural reasons, above all private law regimes through which corporate actors exercise power over resource distribution and governance in the global economy. Ruggie (2018) also points to the network character of TNCs to grasp the conglomeration of subsidiaries, equity affiliates, and further sub-entities. However, this complexity of supply chain capitalism is not reflected in law, because subsidiaries and affiliates are separate legal entities, and the so-called parent company is liable only to a limited extent. Hence, Ruggie (2018: 320) sees a “fundamental disjuncture between economic reality and legal form,” which renders the piercing of the corporate veil difficult. “The fact that public law (national and international) does not generally encompass the economic unity of the multinational firm is the single most important contextual factor shaping its power, authority, and relative autonomy.” (Ruggie 2018: 321). This legal gap also affects the ability to hold companies liable for human rights abuses.

Another obstacle to such an endeavor can be seen in the human rights concept itself. Human rights originally define the relation between the state as the duty holder and the individuals living on its territory and/or under its jurisdiction as rights holders. Today, a triad of state obligations comprises respect for, protection and fulfillment of all human rights, that is, civil, political, economic, social, and cultural rights. There exists broad consensus of the perception of the existing human rights regime as being radically state-centered, reflecting state steering as the dominant mode of governance after World War II (Donnelly 2003). Here, legitimacy is based on the political authority of states with a positive reference to the UN and international law. However, obedience to duties emerging from human rights treaties and from customary international human rights law is—also owing to Article 2 of the UN Charter—understood as being largely at the discretion of governments. Due most notably to poor sanction mechanisms, the regime is considered to be rather weak.

In accordance with the human rights concept, abuses by enterprises have to be prevented by the state as part of its duty to protect. State obligations are also the prominent path in ILO conventions to bind enterprises to labor norms. However, the regulation of businesses guided by human rights was never an important matter of concern by states, who themselves follow economic interests, e.g. attracting investments or protecting the competitiveness of companies based on their respective territories. Thus, a hard fight was always necessary in order to secure human rights in the context of economic activities, for instance for unions to acquire decent working conditions.

The reluctant attitude of states to bind businesses to human rights was further enhanced by changes due to globalization. Here, features of privatization, flexibilization, and enhancement of market solutions marked the tendency of a partial withdrawal of the state as well as a growing influence and power of TNCs (Cutler et al. 1999). Danielsen (2020) points to the increasing accumulation of power due to the coordination and governance of supply chains by large buyer firms. He recognizes a variety of legal techniques and business practices of buyer firms to exercise governance power in their supply chains over their suppliers and the regulatory capacities of the respective governments of production countries. Accordingly, governments of the Global South are impaired in their ability to regulate and follow their human rights duties due to the power of corporations. This includes private investor-state dispute settlements (ISDS) that are often part of free trade and bilateral investment agreements.

The increased power and authority of corporations as well as more generally transnationalization processes are reflected in new modes of governance (Scholte 2005). Following Ruggie (2014: 5), governance “[…] at whatever level of social organization it occurs, refers to the systems of authoritative norms, rules, institutions, and practices by means of which any collectivity, from the local to the global, manages its common affairs.” This definition covers both traditional state steering and private modes of governance that have gained relevance especially in the field of environmental policies (Levy and Newell 2005), and increasingly also in the area of human rights. As a consequence, an overlapping system of nonstate actor-based governance has emerged beside national and international regulation (Haufler 2003). The appropriate governance architecture to deal with global problems that can no longer be dealt with adequately at the national level has been described by Ruggie as polycentric, with various authorities that exercise power (Ruggie 2004, 2014). He considers such a polycentric governance approach as being vital to close the so-called governance gaps above all in the global economy and to meet global challenges such as climate change. To him, integrated regimes become more and more unattainable, because of an increasing complexity and tendencies of fragmentation. He perceives the UNGPs as a prime example of successful polycentric global governance (Ruggie 2014).

### Multi-Stakeholder Initiatives as Transnational Governance

One form of private governance that has become prominent in the global economy is multi-stakeholder initiatives, which are also discussed using the term private regulatory initiatives (PRIs) (Danielsen 2020). Here, the claim to cooperation is in the foreground. This is understood as being a vital means of appropriate governance, and cooperation is also considered to be an important source of legitimacy. Especially, business-civil society collaboration in multi-stakeholder initiatives, which is also referred to as civil regulation (Vogel 2005; Utting 2002), has become a prominent mode of transnational governance. Luc Fransen (2012: 166) defines such multi-stakeholder initiatives as “[…] a universe of initiatives in which the expertise, skills and finance of non-profit and for-profit organizations are pooled.”. Frequently, these initiatives govern social and environmental standards of production across country borders. They have become important in various sectors of the global economy, e.g. in the garment industry, the Social Accountability International (SAI), and the Fair Wear Foundation (FWF). Examples in other sectors are the Forest Stewardship Council (FSC) and the Roundtable on Sustainable Palm Oil (RSPO).

Ruggie emphasizes the relevance of multi-stakeholder initiatives, including in the context of polycentric governance. This view has influenced his work as the UN Special Representative of the Secretary General on business and human rights. Especially the report of 2007 on mapping international standards of responsibility and accountability for corporate acts reflects Ruggie’s general approach to multi-stakeholder initiatives as an appropriate form of governance under conditions of economic globalization: “[..], recognizing that some business and human rights challenges require multi-stakeholder responses, they allocate shared responsibilities and establish mutual accountability mechanisms within complex collaborative networks. These can include any combination of host and home States, corporations, civil society actors, industry associations, international institutions and investors groups. These hybrids seek to enhance the responsibility and accountability of States and corporations alike by means of operational standards and procedures for firms.” (HRC 2007: 17 (53., 54.))

Multi-stakeholder initiatives reflect the increasingly prominent role of companies in global governance, and are particularly supported by the private sector. Accordingly, the World Economic Forum (WEF 2010: 7) emphasizes the necessity of “[…] [r]edefining the international system as constituting a wider, multifaceted system of global cooperation in which intergovernmental legal frameworks and institutions are embedded as a core, but not the sole and sometimes not the most crucial component […]”. This so-called cooperative or partnership approach is also reflected in various types of co-regulation that have emerged at UN level such as the Global Compact and most recently the signing of a Strategic Partnership Framework between the WEF and the UN.

In spite of such appreciation, multi-stakeholder initiatives are also criticized as being part of the neoliberal course of globalization, expressing tendencies of privatization and enhancing market solutions. They not only provide governments with arguments to withdraw from their regulatory responsibilities, but also are perceived as door-openers for the private sector to engage in governance efforts that exceed mere lobbying. Critics understand this shift from government to governance as the economization of authority (Shamir 2010: 535). Others see a risk that non-governmental organizations (NGOs) may become contained in multi-stakeholder initiatives. The fear is that the high moral reputation of NGOs as agenda setters and as watch-dogs of states and the private sector may predominantly be used to legitimize the policies and behavior of the latter two (Martens 2007). This fear is enhanced by an immanent power asymmetry of the participating stakeholders due to different resources and influence. While civil society as the so-called norm entrepreneurs (Florini 1996) may figure above all as source of legitimacy (Levy and Newell 2005), the ultimate authority in discourse and decision-making in such initiatives may be on the business side (Fransen 2012). Despite many reservations and criticisms, Danielsen (2020) also sees a more radical potential of such initiatives, when power structures are broken up by means of bottom-up approaches. These include regional cooperation seeking for living wages in neighboring production countries, as in the Asia Floor Wage Initiative. Another example would be worker-driven social responsibility models that question the paradigm of economic growth.

### Legitimacy and Private Modes of Governance

Even this brief discussion of multi-stakeholder initiatives indicates that—unlike state legitimacy, which is undisputed in principle—private modes of governance lack an unchallenged source of legitimacy. Given the absence of state authority, important norms of legitimacy in the context of global governance are considered to be efficiency, transparency, inclusion, and accountability (Dingwerth and Weise 2012: 111) as well as deliberation (Habermas 1992). Similarly, Luc Fransen (2012) conceives inclusiveness, expertise-based effectiveness, and procedural fairness as important criteria for the legitimacy of multi-stakeholder initiatives.

The unclear source of legitimacy may pose a challenge for participating actors. Thus, NGOs may not wish to make their participation in specific initiatives too public, as they are afraid of being deemed too close to companies. In contrast, companies may consider the participation in multi-stakeholder initiatives as a kind of legitimation for their business activities. This seems especially important as scandals such as the exhaust gas affair and campaigns of civil society organizations have contributed to an overall crisis of legitimacy for economic globalization. Companies try to react to the loss of trust. One example is the business commitment to Corporate Social Responsibility (CSR) in the early 1990s. A dispute over legitimacy is also evident in the field of business and human rights. For example, as will become clear below, Ruggie has always been keen to see the UNGPs gain legitimacy through broad support.

Mark Suchman (1995: 574) emphasizes the significance of consent in his definition of legitimacy as a “[…] perception or assumption that the actions of an entity are desirable, proper, or appropriate within some socially constructed system of norms, values, beliefs, and definitions.” Unlike Suchman, Mlada Bukovansky (2002: 211) focusses on legitimacy as a dimension of power, being the product “[…] of cultural discourses and strategic struggles […],” a feature that is also typical in the discourse on business and human rights. Ian Clark (2007) on the other hand looks at international legitimacy as a contestation between international society (= states) and world society (= civil society). While he emphasizes that “[…] the major way that social norms come to be ‘institutionalized’ is through forms of state regulation, often international” (Clark 2007: 5), he also acknowledges the influence of civil society on the states’ agenda. Clark discusses the interdependencies between state decisions and civil society influence looking at several historic themes such as the abolition of slave trade in the nineteenth century and the inclusion of human rights in the UN Charter in 1945. His list of international topics might be extended to the subject of business and human rights, which was initiated by civil society pressure and later taken up by governments. The present negotiations of a treaty also reveal this interplay between states and civil society.

Moreover, human rights as recognized international norms provide a further immanent source of legitimacy which is worth striving for in the context of business and human rights. Thus, for states, the positive reference to human rights supports “[…] the view that the sovereign state remains the appropriate actor for guaranteeing the rights and freedoms agreed by states.” (Evans 2011: 115). Similarly, the claim of private actors such as businesses and civil society organizations to support human rights and to act in accordance with them constitutes an important source of their legitimacy. This value of human rights to businesses, resulting in greater trust and a stronger social license to operate, has often been accentuated in support of the UNGPs.^5^

The following short section on content and reach of the UNGPs and the RDLBI will offer some insight in the commonalities and differences of the two instruments.

## Content of the UNGPs and the RDLBI

The UNGPs follow a *Policy Framework* involving three pillars—*Protect, Respect, and Remedy*—as presented by Ruggie in 2008 (HRC 2008):

1. The *State Duty to Protect* against human rights abuses by third parties, including business enterprises, through appropriate policies, regulation, and adjudication;
2. The *Corporate Responsibility to Respect Human Rights*, meaning that businesses should act with due diligence to avoid infringing on the rights of others and to address adverse impacts in which they are involved;
3. The need for greater *Access to Remedy* for victims of corporate-related abuse, both judicial and non-judicial.

This design incorporating three pillars with the state duty to protect and the corporate responsibility for human rights as interdependent forms of governance reflects the overlapping system of state and private governance that Virginia Haufler (2003) referred to. However, the specifications on the state duty to protect in the UNGPs concentrate on what states should do and are scarcely in the form of clear obligations. As in pillar two which is based on the voluntary commitment of businesses, the overall governance proposals presented reflect the soft law approach of the UNGPs. This is partly counteracted by pillar three, “Access to Remedy,” which is intended to strengthen the rights of victims of corporate human rights abuses, including through legal measures.

The RDLBI was presented in July 2019, and is based on older proposals that had been discussed at earlier sessions of the OEIGWG. Its content largely builds on the UNGPs. While direct obligations of businesses are avoided, the classic international law position emphasizing the states’ obligations to (legally) regulate companies prevails. Section II constitutes the core of the treaty draft with the focus on two topics, namely rights of victims and their access to remedy as well as prevention in the form of a mandatory corporate human rights due diligence. This approach is strengthened through legal liability in the case of human rights abuses. Thus, the draft displays a clear human rights approach, emphasizing the rights and the protection of victims.

As suggested above, the two instruments mirror different views about the adequate mode of governance for implementing the corporate responsibility for human rights. Thus, the UNGPs reflect Ruggie’s conviction of not traveling the treaty road (Ruggie 2008), and are designed as a soft law instrument. Accordingly, Principle 12 of the Guiding Principles takes the view that “[t]he responsibility of business enterprises to respect human rights is distinct from issues of legal liability and enforcement, which remain defined largely by national law provisions in relevant jurisdictions.” In contrast, the RDLBI emphasizes the need for a legally binding instrument, because “[…] human rights ought to bind all centres of power in society” as Deva and Bilchitz (2014: 2), two major international law proponents for a treaty, have stated in a response to Ruggie’s comment on their book “Human Rights Obligations of Business: Beyond the Corporate Responsibility to Respect?”

Independent of this fundamental difference, the two instruments reveal important similarities, above all with regard to corporate human rights due diligence. This concept was first introduced in pillar two of the Policy Framework and the UNGPs; it proposes a process of due diligence, asking companies to identify, prevent, mitigate, and account for adverse human rights impacts of their own business activities, and also of their business partners. This introduction of human rights due diligence has been praised as a novelty in overcoming mere CSR policies. The concept is familiar to businesses and applied in the context of various activities such as investments and mergers. Ruggie (2014: 14) chose this approach consciously, stating that his “[…] aim was to prescribe practical ways of integrating human rights concerns within enterprise risk management systems.” Therefore, the concept of a corporate human rights due diligence is familiar to business in principle, and it is unsurprising that it encountered approval by the business community. However, international human rights law experts have criticized Ruggie’s intention. Olivier de Schutter (2013: xviii) has warned that “[…] substantive choices may hide behind terminological matters. For instance, mentioning ‘impacts’ rather than ‘violations’ reveals a shift from a legal to a managerial conception of the responsibility of business […].” From a political science perspective, the concept of corporate human rights due diligence is also seen critically as being part of a tendency towards a privatization of human rights (Scheper 2019) and an increasing corporate power of definition in the field of human rights (Felice de 2015).

Such concerns are not considered in the design of the RDLBI. Instead, a major commonality between the UNGPs and the draft versions of the international treaty lies in the elaboration of the corporate responsibility to respect human rights based on due diligence as laid down in the UNGPs. Clearly, the RDLBI builds upon the UNGPs, and without the latter, the content of the treaty might be different, for example reflecting proposals for more direct human rights obligations of businesses.

## The Process Towards the UNGPs

Such thoughts had been included in predecessor of the UNGPs, the *Norms on the Responsibilities of Transnational Corporations and Other Business Enterprises with Regard to Human Rights* (UN 2003). These so-called UN Norms are important in order to understand the two cases discussed here.

### UN Norms—Catalyst in the Business and Human Rights Discourse

The discourse on business and human rights picked up speed in the early 1990s. Already at this time, the two conflicting paths of regulation of global businesses to respect human rights and environmental standards became obvious. On the one hand, UN Secretary General, Kofi Annan, proposed the Global Compact as a voluntary initiative between the UN and the private sector; it was founded in 2000. On the other hand, the former UN Sub-Commission on the Promotion and Protection of Human Rights had assigned a *Sessional Working Group on the Working Methods and Activities of Transnational Corporations* to elaborate a mandatory code of conduct for TNCs in 1998 (Nowrot 2003).^6^

After the unanimous adoption of the draft version of the UN Norms by the UN Sub-Commission, the document was presented to the then UN Commission on Human Rights in 2003.^7^ Kinley et al. (2007) report that regardless of the fact that the document was only a draft, there was no willingness to discuss or negotiate. Instead, fundamentally conflicting views immediately yielded two hostile blocs: While civil society organizations strongly supported the document, the bulk of the private sector and most governments opposed the document vehemently.

Especially the view of a binding regulation for companies was under scrutiny, because the UN Norms not only emphasized the primary responsibility of states to ensure that companies respect human rights, but also intended to transfer direct obligations to the private sector. Representatives of business associations declined this as a “privatization of human rights” (in Parella 2017: 307). Further criticism referred to provisions of implementation in stating that “[t]ransnational corporations and other business enterprises shall be subject to periodic monitoring and verification by United Nations […].” (UN 2003 (16)). This view was rejected as a breach of fundamental principles of international law because it would perceive companies as subjects under international law and weaken national governments in their control of the private sector (Kinley et al. 2007).

Another topic of dispute that also had effects on the elaboration of the UNGPs was the organization of the process leading to the UN Norms. Following David Weißbrodt, who was head of the sessional working group,^8^ they had put special efforts into the inclusion of civil society organizations as well as TNCs in the elaboration of this code. While Deva (2017: 477) emphasizes the “[…] emerging importance of non-state actors in moulding the contours of international law,” he concedes that there were not “[…] enough opportunities to engage during the drafting process […]” for these actors. Overall, the participation of stakeholders became a contentious issue, and business associations marked the whole process as a failure in terms of sufficient consultation, thus also questioning its legitimacy (Kinley et al. 2007: 34).

In April 2004, the then UN Commission on Human Rights considered the draft document and expressed “[…] its appreciation to the Sub-Commission for the work it has undertaken in preparing the draft norms […]”. The Commission stated that they contained “useful elements and ideas for consideration”. But it did not approve of them, stating that they had “no legal standing” (CHR 2004). Thus, the Commission not only denied legitimacy to the document, but also recommended further engagement with the topic. Welcoming the decision, a representative of the International Chamber of Commerce (ICC) explained: “We’re very pleased with the outcome and more than happy to take part in an open discussion on what business can contribute to promoting human rights” (in Parella 2017: 310). Kishanthi Parella (2017) interprets this willingness for discussion as an indirect or penumbra effect of the failed negotiations meant to express the willingness of the private sector to assume responsibility.

### Nomination of John G. Ruggie as SRSG

One consequence of the highly confrontational dispute over the UN Norms was the nomination of Harvard professor John G. Ruggie as the *UN Special Representative of the Secretary-General on human rights and transnational corporations and other business enterprises* (SRSG) in 2005. From the perspective of Susan Ariel Aaronson and Ian Higham (2013: 337f), “Ruggie was a shrewd choice, as he was close to policymakers, NGOs, and business leaders, and he was also the architect of the UN Global Compact, an international initiative to promote globally responsible business behavior.” Especially, the latter raised reservations among civil society organizations, as they feared that Ruggie might transfer the voluntary design of the Global Compact to the topic of business and human rights. This concern was not unsubstantiated, as the Global Compact also reflects Ruggie’s theoretical approach to global governance with businesses as potential problem solvers and important actors in global governance.

Considering the antagonistic outset after the refusal of the UN Norms, one major challenge of Ruggie’s work as SRSG was how to bridge conflicting views and positions. Ruggie himself perceived his “mandate as a means to move beyond the stalemate” created by the debate on the UN Norms (CHR 2006: 14 (55)). CSR researcher Bryan Horrigan (2010: 324) sees one of the strengths of Ruggie’s work as having unblocked the polarized dispute. Ruggie’s efforts towards consensus building can themselves be seen as an important source of legitimacy: All stakeholders, even civil society organizations in spite of some persisting reservations, supported the endeavor.

However, in spite of his efforts to open up the discussion among the different stakeholders, the Special Representative revealed a downright negative view towards the UN Norms from the beginning. Thus, he characterized the process as a “train wreck” and declared the Norms as “dead” (in Kinley et al. 2007: 31). The so-called interim report, published in February 2006, also reveals Ruggie’s dismissal of the Norms. He classified them as “doctrinal excesses” with “[…] exaggerated legal claims and conceptual ambiguities [that] created confusion and doubt even among many mainstream international lawyers […].” (CHR 2006: 14 (59)). The language Ruggie uses here suggests more than a refusal based on objective grounds. Terms like “doctrinal excesses,” “exaggerated legal claims,” or “doubt among mainstream international lawyers” tend to disqualify and marginalize the document and its authors. Overall, the SRSG expressed “[…] the view [that] the divisive debate over the Norms obscures rather than illuminates promising areas of consensus and cooperation among business, civil society, governments and international institutions with respect to human rights.” (CHR 2006: 17 (69)).

### The So-called Ruggie Process

The Special Representative’s mandate lasted 6 years from 2005 to 2011 and covered three phases. Ruggie commanded a team of researchers and practitioners to support his work, and also gained the support of international organizations such as the International Finance Corporation (IFC) for funding and expertise. The Ruggie team held forty-one multi-stakeholder meetings on all continents; every document, comment, and meeting report was posted on the website of the Business and Human Rights Resource Centre (BHRRC).^9^

During the first phase (2005–2007), Ruggie was asked to clarify controversial concepts such as “corporate sphere of influence” and identify best practices. In the already mentioned interim report, Ruggie outlines the broader context of his mandate, namely globalization, corporate abuses, and existing responses. He also confronts the UN Norms with his own strategy of “principled pragmatism” as the right way forward. As a method, Ruggie chose empirical studies to identify specific challenges in the field of business and human rights, e.g. the situation on the ground in different economic sectors. For this purpose, Ruggie and his team undertook five surveys investigating the attitude and views of companies regarding their human rights policies (Aaronson and Higham 2013: 341f). Beyond field research, he also refers to different legal sources and to the knowledge of scientific institutions as well as projects with business associations in order to identify “[…] effective ways for companies to deal with dilemma situations encountered in ‘weak governance zones’.” (CHR 2006: 18 (75)).

The second phase up to 2008 culminated in the presentation of the three-pillar *Policy Framework—Protect, Respect, and Remedy* (HRC 2008). Ruggie’s aim was to reflect the complexities and dynamics of globalization and “[…] to reduce or compensate for the governance gaps created by globalization, because they permit corporate-related human rights harm to occur even where none may be intended” (HRC 2008: 5 (11)). Public consultations were organized in the internet under the motto “We need your views!” In addition, a legal analysis of existing treaties and procedures under international law was undertaken, and Ruggie concluded that there was “little evidence to support the claim that companies have direct human rights obligations under international law.”^10^

In the next phase, Ruggie had another 3 years for the operationalization of the Policy Framework, which resulted in the UNGPs in 2011. The Ruggie team asked for public comments on the UNGPs. However, only ninety submissions were handed in, above all from academics and activists (Aaronson and Higham 2013: 345). Altogether, Ruggie (2014: 5) proudly states that this “[…] was the first time that the UN adopted a set of standards on the subject of business and human rights; and […] the only time the commission or council endorsed a normative text on any subject that governments did not negotiate themselves.”

### The UNGPs—a Model of Global Governance

The process towards and design of the Policy Framework and—building on this—the UNGPs reflect Ruggie’s theoretical thinking as prominent advocate of global governance, as he elaborates in the article “Global Governance and ‘New Governance Theory’: Lessons from Business and Human Rights” (Ruggie 2014). Aaronson and Higham (2013: 333) see Ruggie’s approach as the way to success: “[…] the process of developing the [UNGPs] was a model of transparent, inclusive twenty-first century governance, although the public is generally unaware of the issue or the new policy […].” Similarly, international law professor Larry Catá Backer perceives the process towards the UN Policy Framework as an innovative approach that is able to re-embed the political, economic, and social systems. “[…], this framework seeks inter-systemic harmonization that is socially sustainable, and thus stable.” (Backer 2012: 79). To him, those emphasizing the state duty to protect are conventional and more or less turned backwards (Backer 2012: 80).

This appraisal of the UNGPs partly rests upon the broad global multi-stakeholder process with “[…] nearly fifty international consultations on five continents, numerous site visits to individual firms and local communities, extensive research, and pilot projects to road test key proposals.” (Ruggie 2014: 5). Collaboration and consensus are seen here as prominent sources of legitimacy. In addition, Ruggie (2014: 5) also emphasizes the independence of the process. He underlines that his appointment as Special Representative was an “an unpaid position,” and that he managed to attract “[…] voluntary contributions from supportive governments structured as research grants to Harvard’s Kennedy School of Government^11^ […].” (Ruggie 2014: 16, endnote 13). Two dozen corporate law firms conducted a survey of the relationship between corporate law and human rights in thirty-nine jurisdictions around the world on a pro bono basis (Ruggie 2014: 14). To what extent Ruggie’s project was also financially supported by the private sector remains unclear. All in all, Ruggie was in a well-equipped position, e.g. to initiate diverse research projects on the human rights situation in various economic sectors. This empirical work offers important insights into the structure of the global economy until today. However, this extensive work did not lead the Ruggie team to onsite visits to places where corporate human rights abuses had occurred. Thus, there was no direct exchange with people affected.

Ruggie (2014) himself underlines the success of the multi-stakeholder process leading to the UNGPs. He thereby puts the need for legitimacy into the foreground when speaking of the broad approval for the UNGPs in July 2011 as “thick stakeholder consensus,” hoping that this was normatively superior in securing compliance compared to the “thin state consent” in the context of international law.

Beyond some critical voices, the overall approval of the UNGPs was overwhelming when they were adopted in 2011. Aaronson and Higham (2013: 345) acknowledge the overall process as a success: “In sum, over a relatively short period, Ruggie and his team created a workable approach for firms to evaluate, monitor, and address human rights that gained international approval.” They consider the process as inclusive and transparent. However, the two authors only appreciate the usefulness for businesses, and fail to discuss critical views that the UNGPs might enhance the privatization of human rights. In sharp contrast to Aaronson and Higham, Deva questions the meaningfulness of the stakeholder approach of the Ruggie process. He (Deva 2013: 85; highlighted in original) makes the criticism that critical voices of NGOs and scholars were not well-documented or taken up, in contrast to favorable views, which “[…] were splashed all over the media to paint dissenting voices as an insignificant minority; […]. The advice of all stakeholders, including NGOs, was sought and valued, but only within the framework set by the SRSG. […], the *core* of the Ruggie project was not open for change and […] hardly changed despite extensive consultations.” Consequently, Deva (2013: 86) argues that “[…] the SRSG consultations were designed primarily to acquire legitimacy, something which is badly needed when a small group of persons are engaged in the task of international law-making.”

## Bumpy Treaty Road

The 6-year process towards the UNGPs may be described as being intense, smooth, and well equipped. Ruggie succeeded in gaining broad consent for his work as SRSG and for its outcome, the UNGPs; and he wanted to celebrate the thick consensus not only for the UNGPs, but also for the process as an example of successful global governance. Many Western governments, especially from Europe, actively supported the undertaking. The project gained quite a lot of public attention, for example at international conferences that were organized by governments in various European cities such as in Stockholm and Berlin. Moreover, the UNGPs had been promoted in the Global South with projects and funding from development aid of various Western countries. Especially the private sector with its international business associations, among them the IOE, the ICC, and the Business Advisory Committee to the OECD (BIAC) praised the UNGPs. Single companies also got involved and supported Ruggie’s work. These followers of the UNGPs are also those who promote the neoliberal course of the global economy with an emphasis on market solutions, flexibilization, and privatization. In line with this, binding regulation for corporations in the fields of human rights and environmental standards are denied. Rather, the UNGPs are presented as an adequate instrument at bringing the global economy in line with human rights. This is in stark contrast to the prevalence of binding regulation, e.g. by means of corporate law and private courts, when economic interests and the organization of global supply chain capitalism are in the foreground.

In spite of the broad backing, Ruggie’s project did not go so smoothly, because there was reluctance among governments of the Global South, international law experts, and above all civil society organizations. Some critics feared a lack of efficient regulation and impunity in cases of corporate human rights abuse. Some also saw the UNGPs and the design of corporate human rights due diligence as a management tool as an expression of corporate capture.

However, the supporters of a legally binding instrument are not as powerful and financially strong as those in favor of the UNGPs. Thus, compared to the UNGPs, the process towards a binding instrument is much more conflictive and not well equipped. In contrast to the publicity of the so-called Ruggie process, the procedure for a binding instrument on business and human rights has emerged as fundamentally different, and less glamourous. Public awareness is predominantly created through the activities of civil society organizations.

In the beginning, the working group and also the chairmanship of Ecuador had been contested above all by the EU as one important leader of the Global North, challenging the mandate and the financing of the OEIGWG. Especially the private sector questioned the necessity of a treaty in principle. Regardless of many attempts of delegitimization, the working group has recognized UN procedures at its disposal: The UN and the states as negotiators constitute accepted authorities, and thus at least at a formal level the OEIGWG possesses legitimacy. It seems that the Chair uses these resources thoughtfully. The following section will present the work of the OEIGWG since 2014 and discuss the conflict lines along different actors and controversial issues.

### The OEIGWG Since 2014

Ruggie’s hope of a thick stakeholder consensus for the UNGPs turned out to be mistaken, because—as described above—already at the time of passing the instrument in 2011, there was dissent among those states who were then members of the HRC.^12^ As a consequence, the HRC did not take a vote, but merely unanimously adopted the UNGPs. The reason for this procedure was that Ecuador and South Africa would have abstained from voting, and a non-unanimous approval in the HRC had to be avoided in order to emphasize accordance (López 2013: 71) and thus also the legitimacy of the UNGPs. In the following, the two states were joined by more than 80 governments from the Global South in their request for a binding instrument to regulate TNCs in the global economy.^13^

In June 2014, the open-ended intergovernmental working group on transnational corporations and other business enterprises with respect to human rights (OEIGWG) was mandated by the HRC in its resolution 26/9 “to elaborate an international legally binding instrument on transnational corporations and other business enterprises with respect to human rights.” (HRC 2014). The Chair-Rapporteur of the OEIGWG from Ecuador was elected during the first session.

From 2015 until 2019, the OEIGWG conducted five official sessions in Geneva, mostly in October. State participation at these gatherings varied from 60 registered governments at the first session, with a peak of 99 at the third session to 89 states at the fifth session. There were also informal discussions between the formal meetings, which the chair repeatedly referred to, probably in order to indicate that as for the UNGPs, there was broad consultation. The first two sessions were scheduled for consultation regarding the content, reach, and character of the treaty, which is summarized in the first document of the OEIGWG, the so-called Elements. The following sessions were devoted to negotiations of diverse draft text that had been presented by the Chair-Rapporteur in advance:

- ELEMENTS FOR THE DRAFT LEGALLY BINDING INSTRUMENT ON TRANSNATIONAL CORPORATIONS AND OTHER BUSINESS ENTERPRISES WITH RESPECT TO HUMAN RIGHTS, presented in September 2017, and discussed at the third session of the OEIGWG in October 2017^14^;
- ZERO DRAFT: LEGALLY BINDING INSTRUMENT TO REGULATE, IN INTERNATIONAL HUMAN RIGHTS LAW, THE ACTIVITIES OF TRANSNATIONAL CORPORATIONS AND OTHER BUSINESS ENTERPRISES, presented in July 2018 as well as an Optional Protocol to the Zero Draft covering “National Implementation Mechanisms” presented in September 2018, and negotiated at the fourth session of the OEIGWG in October 2018^15^;
- REVISED DRAFT: LEGALLY BINDING INSTRUMENT TO REGULATE, IN INTERNATIONAL HUMAN RIGHTS LAW, THE ACTIVITIES OF TRANSNATIONAL CORPORATIONS AND OTHER BUSINESS ENTERPRISES (RDLBI), presented in July 2019, and covered at the fifth session of the OEIGWG in October 2019.^16^

Each session of the OEIGWG was followed by an official report presented by the Chair-Rapporteur.^17^ Mostly, the inputs of delegations and other stakeholders documented in these reports are anonymous, and thus cannot be assigned to specific countries or organizations. However, the panelists of each session are listed by name and respective affiliation. They are representatives of human rights relevant UN institutions and other international organizations, international law experts from universities and law firms, NGOs, unions, and others from the business sector, coming from the Global North as well as the Global South. In contrast to the continuous absence of the US government, many panelists are from the USA. Overall, the panelists represent all constituencies in the debate on a legally binding instrument on business and human rights in the global economy. It is therefore proposed that—in addition to the presence and negotiation of states as well as the UN organizing the event—the balanced composition of panelists can be considered a source of legitimacy for the process.

### Familiar Conflict Lines

Well-known lines of conflict, which have shaped the discourse on business and human rights for many years, showed themselves once again during the negotiations in Geneva. Thus, similar to the UN Norms, the group of Western industrial countries together with the private sector were in strong opposition to a treaty. However, from the beginning, the endeavor encountered unwavering endorsement from a broad transnational civil society network, asking for a vigorous binding instrument.

The different positions of governments from the Global North and the Global South were also reflected in the voting behavior at the HRC to install the OEIGWG. Only 20 countries voted in favor of the resolution, among them China, India, and Russia as important economic players. Besides 13 abstentions, 14 countries voted against, among them all Western industrial countries that were present at the meeting of the HRC, namely Austria, France, Germany, Ireland, Italy, Japan, the UK, and the USA.^18^ Thus, the home countries of the vast majority of the world’s TNCs were opposed to the resolution. This voting behavior expresses the hostile attitude of the Global North with respect to a binding instrument on business and human rights, and the dismissal continued during the sessions of the OEIGWG. Thus, at first sight, the negotiation process looked like a revival of the North-South conflict of the 1970s when demands for a New Economic World Order were on the international agenda (see also Deva 2017: 478f). However, as the abstentions in the voting process reveal, there was no unanimous support for a binding instrument among the governments of the Global South. Former SRSG Ruggie (2014a: 3f) emphasizes the tight vote, classifying it as weak with only “the thinnest of political mandates” for the treaty sponsors. He further fuels the conflict between the two projects and the struggle for legitimacy by pointing to the unanimous adoption of a resolution to continue the already existing Working Group on the UNGPs at the same session of the HRC.

A strong and specific role in the negotiations is held by the EU, even without a negotiating mandate from the Council of Europe. Due to the absence of the USA at the negotiations, the weight of the EU was even stronger. With its economic power, the EU is an important framer of the neoliberal course of the global economy, for example in free trade and investment agreements where human rights and environmental standards are scarcely covered. At the same time, the EU always has been a strong supporter of the UNGPs, and from the beginning has expressed strong reservations against the treaty project. Thus, during the first four sessions, the EU had put pressure on the Chair from Ecuador with respect to content, especially revisions concerning scope and direct human rights obligations of corporations. In addition to the EU position, the views of individual European states became also apparent: While Germany was among those states revealing a continuous blockade mentality and hiding behind the EU delegation, others, such as Spain, France, and Belgium, took a more pro-active role in the process.

In addition to its own neoliberal orientation and the lobbying of European TNCs, the EU has also been under pressure from European NGOs that are in firm support of an international treaty asking for strong measures of accountability for TNCs. Despite opposing views regarding the treaty, there is also accordance. The EU seems well aware of the significance of civil society organizations in the process, and turned down an attempt of the Chinese delegation to restrict their participation at the negotiations in Geneva. Clark (2007) points to the interaction and interdependence between states and transnational civil society organizations in order to establish “new” norms and, based on these, international legitimacy. This interplay can also be seen with respect to the proceedings in Geneva, when treaty promoters of the Global South look to “civil society power […] to mount pressure on the [..] EU to engage with the treaty process.” (Deva 2017: 479). After the already mentioned revisions of the treaty drafts, the EU delegation gave up its strict blockade strategy at the end of the fifth session, agreeing that a legally binding instrument might have added value.

This presumed yielding may have diverse reasons. There was pressure on the EU that came from the campaign “Rights for People, Rules for Corporations – Stop ISDS” between early 2019 and the beginning of 2020. A broad transnational civil society network that united globalization critics and human rights advocates from 16 countries had started this campaign, and collected nearly 850,000 signatures in European countries. The aim was to combine the request for a binding instrument on business and human rights with the demand that the mechanism of a private investor-state dispute settlement (ISDS) in the context of Free Trade and Bilateral Investment Agreements must be stopped. Such dispute settlement procedures protect the interests of corporations in a one-sided manner, while human rights and environmental concerns of people affected remain unenforceable. With this convergence of two topics, namely human rights in the global economy and trade and investment, the campaign raised a sensitive topic for the EU, aiming at raising awareness of the power asymmetry between TNCs and many states of the Global South as well as people living there. There was a danger that due to this campaign, the consent to the then pending reform of the ISDS and to the passage of diverse free trade agreements could be denied, and criticism of the neoliberal policies of the EU might intensify.

The pressure from civil society campaigning was complemented by civil society participation at the negotiations in Geneva as well as the organization of side events parallel to the official meetings. To attend the sessions of the OEIGWG, stakeholders need to have an accreditation in the form of a consultative status with the Economic and Social Council (ECOSOC) of the UN. Due to this procedure, stakeholder inclusion is less broad compared to the UNGPs. The number of NGOs as well as unions in consultative status with the ECOSOC varied at the meetings of the OEIGWG.^19^

One persistent line of conflict at these meetings has been between civil society organizations and business associations. It is well known that the private sector, associations and single companies alike, was very engaged in the Ruggie process and welcomed the UNGPs as the appropriate instrument to implement the corporate responsibility for human rights. In contrast to this commitment, the attitude towards the OEIGWG and the international treaty is the opposite. As noted above, business organizations repeatedly expressed their rejections of a binding instrument as it would diverge from the accepted approach taken in the UNGPs (HRC 2019: page 5, 11).^20^ Perhaps bearing in mind the influence of the private sector in the preparation of the UNGPs, at the OEIGWG negotiations, civil society organizations repeatedly warned of the danger of corporate capture in the field of business and human rights. The continued use of this argument may also be taken as an attempt to delegitimize the participation of the private sector and to deny it a role as problem solver. Against this background, some state delegations expressed their concern that the whole process might become “anti-business and that business should have a greater voice in it.” (HRC 2019: page 6, 22). This view was supported by a business organization requesting that the private sector should “be consulted more fully, noting that there had been a lack of meaningful discussion with its members on important substantive issues.” Also, others have warned that businesses need to be better included in order to have a successful treaty process (Ruggie 2014a; Deva 2017: 479).

### Controversial Issues

The already mentioned presumed yielding at the end of the fifth session may also be partly attributed to the fact that the EU had prevailed in the negotiations in Geneva regarding controversial issues. Conflictive topics during the five sessions until 2019 were the appropriate relationship between the UNGPs and an international treaty, the position of such an instrument in international law, and the scope and the overall shaping of the corporate responsibility for human rights.

Especially at the first sessions, interventions regarding the UNGPs were not meant as proposals to make linkages between the two instruments, but were statements to undermine the need for a treaty. Above all, the EU underlined the progress already made in implementing the UNGPs, pointing to the relevance of National Action Plans (NAPs). Another contentious issue that dominated the discussions at the various sessions referred to scope. This involved the question whether only TNCs (as favored above all by the Group of African States) or all businesses (as requested by the EU) should be the target of the treaty. This persistent controversy was already evident in the 1970s in the dispute over an international code of conduct for TNCs.

Among all the draft documents presented by the Chair-Rapporteur, the “elements document” officially discussed at the third session was the most controversial one. Not surprisingly, 99 states were registered for this session, which was the highest state participation in the process so far. The document not only referred—in addition to the UNGPs—to the UN Norms, but also imposed direct obligations on companies, and under the topic of Principles (Article 1.2) emphasized “the primacy of human rights obligations over trade and investment agreements”. All these very controversial issues were particularly rebuffed by representatives of the Global North. Moreover, further tensions with respect to procedures became apparent, as a few days earlier a pre-meeting had taken place, which not everyone could attend. Criticism of this procedure was countered by the Chair-Rapporteur from Ecuador referring to the legitimacy of the negotiation process by pointing to the more than 200 meetings held since 2014 involving multiple stakeholders. Like some state delegations, business organizations expressed concern that the “elements document” had been published only 3 weeks before the session, not allowing for sufficient time to analyze and formulate official positions on the content. Thus, the third session was dominated by conflicts regarding not only content but also procedure, questioning the objectivity and transparency of the process.

## Conclusion

The two instruments for business and human rights, the UNGPs and the RDLBI, have emerged from two fundamentally distinct processes, with states, businesses, and civil society organizations taking sometimes different roles and building different alliances. The treaty draft builds on the UNGPs, especially with respect to the design of the corporate human rights due diligence. Nevertheless, the divide between a voluntary and binding path is mirrored in the two approaches.

The initial proposal was to look at the two projects as a struggle over the power of definition and legitimacy in the field of business and human rights:

Guided by the theoretical thinking of Ruggie (2004, 2014), the UNGPs emerged from a broad multi-stakeholder process as a form of transnational governance. The procedure may be characterized as broad consultations rather than open negotiations. The strong engagement of the private sector, associations, and single businesses, underlines Ruggie’s view of the need for all relevant actors to collaborate, and is reflected in the content of the UNGPs. Enterprises are perceived as problem-solvers and considering the content of the UNGPs even as norm entrepreneurs. Above all, Western industrial states supported the process through various means, e.g. financing, and also by globally spreading the ideas of the UNGPs.

The process towards the RDLBI follows UN rules. While states have been negotiating the treaty drafts, opposing positions have become apparent. Overall, governments from Western industrial countries and above all the EU have been reluctant to accept the treaty. Instead, these actors have exercised their power, and induced the Chair to undertake substantial changes in content. This is especially true regarding the scope and position of the treaty in international law. Thus, outcome is based on power, political decisions, and interests. In spite of significant alterations up to now, the treaty is important, as the two instruments may complement each other by strengthening the corporate responsibility for human rights through legal liability.

Both processes reflect the struggle for legitimacy. Several statements of Ruggie about the approval of the UNGPs in the HRC reveal how important he considers legitimacy for the success of the instrument. With regard to the RDLBI, the negotiation of states and the UN no longer seem to be sufficient sources of legitimacy. In order to counter accusations, the Chair of the OEIGWG tries to extend transparency and inclusion by means of informal consultations. Attempts of delegitimization by the EU and European states were especially apparent at the time of the installation of the OEIGWG. The private sector continuously deplores the lack of transparency and inclusion, while civil society organizations warn of a corporate capture. In this contested situation, Deva (2017: 485f) warns against excluding the private sector from the negotiations, and Ruggie (2014a: 7) proposes softening the consultative status with the ECOSOC and include individual companies in the negotiations.

While the two processes each contain their own sources of legitimacy, both have revealed shortcomings in this respect. With regard to a multi-stakeholder process, it seems important to define more precisely how meaningful consultations are to be designed. The negotiation process at the UN may require more flexibility and reforms for broader participation.

The OEIGWG’s sixth session took place from 26 to 30 October 2020 in Geneva; due to the COVID-19 pandemic and also to the financial crisis of the UN, the conference was subject to special restrictions (HRC 2021). Nevertheless, 66 states had registered, and 82 civil society organizations participated in the negotiations. In addition to judicial concerns such as mutual legal assistance, major conflicts arose regarding the treaty’s relation to trade and investment obligations of states as well as the institutional features of the treaty, including financing. At this sixth meeting, some readiness to compromise also became apparent. Thus, the EU underlined the relevance of a treaty for creating a level playing field, and even the IOE declared its willingness to further lobby important states.

Nevertheless, because of the lack of “thick consensus” among states, the pressure of civil society organizations seems vital in order to reach a treaty that brings the rights of victims to the fore and covers the legal liability of enterprises in the global economy. It seems clear that further compromise is necessary, because the dismissal of powerful countries in the treaty negotiations could cause the project to fail or become meaningless, like the code of conduct for TNCs in the 1970s. Civil society organizations can play an important role in exerting pressure for the treaty. It remains to be seen how successful this will be. Some civil society organizations already consider that the text has become too diluted.

## Footnotes

1 Human Rights Watch (2011): UN Human Rights Council: Weak Stance on Business Standards, http://www.hrw.org/node/99908. Accessed 26 May 2020

2 https://www.un.org/en/charter-united-nations/. Accessed 8 December 2019

3 The terms MNE and TNC are used here synonymously.

4 https://unctad.org/en/Docs/diaeia20092_en.pdf. Accessed 7 October 2020

5 E. g., https://www.ungpreporting.org/resources/the-ungps/. Accessed 14 May 2020

6 An earlier involvement with the subject of human rights and TNCs at UN level goes back to the 1970s. In the context of the demand of developing countries for a New International Economic Order (NIEO) to better consider the interests of these countries vis-à-vis Western industrialized countries, the Centre on Transnational Corporations was established in 1975. It was mandated with the elaboration of a Draft Code of Conduct on Transnational Corporations. However, the project failed above all due to fundamental resistance from Western industrial countries.

7 https://www.business-humanrights.org/en/united-nations-sub-commission-norms-on-business-human-rights-explanatory-materials. Accessed 22 March 2020

8 http://library.fes.de/pdf-files/bueros/genf/50029.pdf. Accessed 22 March 2020

9 BHRRC, UN Secretary-General’s Special Representative on business and human rights, *available at* https://www.business-humanrights.org/en/un-secretary-generals-special-representative-on-business-human-rights

10 John Ruggie, Prepared Remarks at Clifford Chance 2 (19 Feb. 2007), *available at* http://www.reports-and-materials.org/Ruggie-remarks-Clifford-Chance-19-Feb-2007.pdf. Accessed 27 March 2020

11 Ruggie’s position as a professor is at this Harvard school.

12 The 47 Member States of the HRC are elected by the General Assembly of the UN, and serve for a period of 3 years. Membership is based on equitable geographical distribution. The Bureau of the Council consists of five persons—one President and four Vice-presidents—representing the five regional groups. In 2011, these were Argentina, India, Nigeria, and Russia. Norway had the presidency and “[…] made the endorsement of the Guiding Principles a […] national priority.” (Ruggie 2013: xiii).

13 https://www.business-humanrights.org/sites/default/files/media/documents/statement-unhrc-legally-binding.pdf. Accessed 23 May 2020

14 https://www.ohchr.org/Documents/HRBodies/HRCouncil/WGTransCorp/Session3/LegallyBindingInstrumentTNCs_OBEs.pdf. Accessed 19 November 2020

15 https://www.ohchr.org/documents/hrbodies/hrcouncil/wgtranscorp/session3/draftlbi.pdf. Accessed 19 November 2020

16 https://www.ohchr.org/Documents/HRBodies/HRCouncil/WGTransCorp/OEIGWG_RevisedDraft_LBI.pdf. Accessed 19 November 2020

17 The reports are available at the website of the Office of the High Commissioner for Human Rights (OHCHR). https://ap.ohchr.org/documents/dpage_e.aspx?si=A/HRC/34/47. Accessed 23 November 2019

18 The 13 abstentions came from Argentina, Botswana, Brazil, Chile, Costa Rica, Gabon, Kuwait, Maldives, Mexico, Peru, Saudi Arabia, Sierra Leone, and the United Arab Emirates.

19 Among them was also the International Organisation of Employers (IOE).

20 In contrast to business associations, individual companies are in favor of a treaty.
